# Supplementary material for: Communities on the Move: Pedestrian-Oriented Zoning as a Facilitator of Adult Active Travel to Work in the United States
Source: Front Public Health. 2016 Apr 18;4:71. doi: 10.3389/fpubh.2016.00071 (PMC4834572; doi:10.3389/fpubh.2016.00071)
Supplement: Supplementary file 1 [file Data_Sheet_1.PDF]

NCI Code Reform Project: Policy Coding Instrument

A. Community Identification Information

FIPS15: __ __ __ __ __ __ __ __ __ __ __ __ __ __ __

Summary Level Code: ___ ___ ___

Place Name:

County Name:

State Name:

Keep Level:

B. Coder and Zoning Code Information

Coder ID Number: 1 0 ___ ___

Coding Date: ___ ___ / ___ ___ / 2 0 ___ ___

Community Type	
County	1
Place	2

Zoning Code Status	
Zoning code exists	1
No zoning code (verified)	0
Missing zoning code (non-responder)	9

If "Zoning code exists" is selected, continue to next column

B. Coder and Zoning Code Information (Cont.)

Policies Coded	
County	1
Place	2
Place and County	3

Zoning Code Adoption Date

___ ___ / ___ ___ / ___ ___ ___ ___

Zoning Code Most Recent Amendment Date

___ ___ / ___ ___ / ___ ___ ___ ___

Zoning Code Source(s)	Y	N
Online code publisher	1	0
Other code publisher	1	0
Community website	1	0
Planning/Zoning Office website	1	0
Community mail/email	1	0
Other (specify):	1	0

Zoning Code Type(s)	Y	N
Traditional/Euclidean	1	0
Code Reform	1	0
Unified Development Code (UDC)	1	0

If "Code Reform" is selected, provide dates below

Code Reform Adoption Date

___ ___ / ___ ___ / ___ ___ ___ ___

Code Reform Most Recent Amendment Date

___ ___ / ___ ___ / ___ ___ ___ ___

If "Code Reform" is selected, continue to next column

B. Coder and Zoning Code Information (Cont.)

Code Reform Type	
SmartCode*	1
Full Form-Based Code (non-SmartCode)	2
Code Reform District(s)/Regulations Only	3

*Full SC?	
Y	N
1	0

Code Reform District(s)/Regulations Present in Overall Zoning Code	Present?		Overlay?		Optional?	
	Y	N	Y	N	Y	N
Form-Based District(s)/Regulations	1	0	1	0	1	0
Transect-Based Zone(s)/District(s)/Regulations	1	0	1	0	1	0
New Urbanist District(s)/Regulations	1	0	1	0	1	0
Pedestrian-Oriented Development (POD) District(s)/Regulations	1	0	1	0	1	0
Transit-Oriented Development (TOD) District(s)/Regulations	1	0	1	0	1	0
Traditional Neighborhood Development (TND) District(s)/Regulations	1	0	1	0	1	0
Other Code Reform District(s)/Regulations (specify):	1	0	1	0	1	0

ZONING DISTRICT CATEGORY DEFINITIONS

Code Reform = Form-Based, Transect-Based, New Urbanist, POD, TOD, or TND districts/regulations

Commercial = Commercial, Downtown, or Highway districts

Mixed Use = Mixed Use districts

Park, Rec, Open Space = Park, Recreation, or Open Space districts

Planned Unit Development (PUD) = all PUD (residential, commercial, and/or mixed) regulations

Public, Civic, Gov't = Public, Civic, Government, School, or Institutional districts

Residential = Residential districts

General Zoning Provisions = Overall Zoning Code or UDC (non-Code Reform)

STRENGTH OF PROVISION DEFINITIONS

Req (Required) = strongest provision found in district category has strength of required

Enc (Encouraged) = strongest provision found in district category has strength of encouraged

No = strongest provision found in district category has no strength

TYPE OF USE DEFINITIONS

Allow (Allowed)= permitted, conditional, or accessory use

None= use not specified

[**Prohib** (Prohibited)= prohibited use; *only applicable to mixed use markers*]

[**Mixed**= both allowed and prohibited uses for districts within category; *only applicable to mixed use markers*]

Copyright © 2013 The Board of Trustees of the University of Illinois

C. Zoning District Category Coding

ZONING DISTRICT CATEGORIES PRESENT	Yes	No	Community Districts Coded
**1. CODE REFORM CATEGORY**	1	0	
**2. COMMERCIAL DISTRICTS CATEGORY**	1	0	
**3. MIXED USE DISTRICTS CATEGORY**	1	0	
**4. PARK, REC, OPEN SPACE DISTRICTS CATEGORY**	1	0	
**5. PLANNED UNIT DEVELOPMENT (PUD) CATEGORY***	1	0	
**6. PUBLIC, CIVIC, GOV'T DISTRICTS CATEGORY**	1	0	
**7. RESIDENTIAL DISTRICTS CATEGORY**	1	0	
**8. GENERAL ZONING PROVISIONS**	1	0	

*PUD Type(s)	Y	N
Primarily Commercial	1	0
Primarily Residential	1	0
Mixed	1	0
General or Unknown	1	0

Answer if PUD category is present

Category Present?

Y	N

1. Code Reform Category	Addressed?		Strength			Type of Use				Citation(s)
	Yes	No	Req	Enc	No	Mixed	Allow	Prohib	None	
A. Sidewalks	1	0	2	1	0	-	-	-	-	
B. Crosswalks	1	0	2	1	0	-	-	-	-	
C. Bike-Pedestrian Connectivity	1	0	2	1	0	-	-	-	-	
D. Street Connectivity	1	0	2	1	0	-	-	-	-	
E. Bike Lanes	1	0	2	1	0	-	-	-	-	
F. Bike Parking	1	0	2	1	0	-	-	-	-	
G. Bike-Pedestrian Trails-Paths	1	0	2	1	0	-	1	-	0	
H. Other Walkability	1	0	2	1	0	-	-	-	-	
I. Mixed Use	1	0	2	1	0	2	1	-1	0	
J. Active Recreation	1	0	2	1	0	-	1	-	0	
K. Passive Recreation	1	0	2	1	0	-	1	-	0	

Y	N

2. Commercial Districts Category	Addressed?		Strength			Type of Use				Citation(s)
	Yes	No	Req	Enc	No	Mixed	Allow	Prohib	None	
A. Sidewalks	1	0	2	1	0	-	-	-	-	
B. Crosswalks	1	0	2	1	0	-	-	-	-	
C. Bike-Pedestrian Connectivity	1	0	2	1	0	-	-	-	-	
D. Street Connectivity	1	0	2	1	0	-	-	-	-	
E. Bike Lanes	1	0	2	1	0	-	-	-	-	
F. Bike Parking	1	0	2	1	0	-	-	-	-	
G. Bike-Pedestrian Trails-Paths	1	0	2	1	0	-	1	-	0	
H. Other Walkability	1	0	2	1	0	-	-	-	-	
I. Mixed Use	1	0	2	1	0	2	1	-1	0	
J. Active Recreation	1	0	2	1	0	-	1	-	0	
K. Passive Recreation	1	0	2	1	0	-	1	-	0	

Y	N

3. Mixed Use Districts Category	Addressed?		Strength			Type of Use				Citation(s)
	Yes	No	Req	Enc	No	Mixed	Allow	Prohib	None	
A. Sidewalks	1	0	2	1	0	-	-	-	-	
B. Crosswalks	1	0	2	1	0	-	-	-	-	
C. Bike-Pedestrian Connectivity	1	0	2	1	0	-	-	-	-	
D. Street Connectivity	1	0	2	1	0	-	-	-	-	
E. Bike Lanes	1	0	2	1	0	-	-	-	-	
F. Bike Parking	1	0	2	1	0	-	-	-	-	
G. Bike-Pedestrian Trails-Paths	1	0	2	1	0	-	1	-	0	
H. Other Walkability	1	0	2	1	0	-	-	-	-	
I. Mixed Use	1	0	2	1	0	2	1	-1	0	
J. Active Recreation	1	0	2	1	0	-	1	-	0	
K. Passive Recreation	1	0	2	1	0	-	1	-	0	

Copyright © 2013 The Board of Trustees of the University of Illinois

Y	N	4. Park, Rec, Open Space Districts Category	Addressed?		Strength			Type of Use				Citation(s)
			Yes	No	Req	Enc	No	Mixed	Allow	Prohib	None	
		A. Sidewalks	1	0	2	1	0	-	-	-	-	
		B. Crosswalks	1	0	2	1	0	-	-	-	-	
		C. Bike-Pedestrian Connectivity	1	0	2	1	0	-	-	-	-	
		D. Street Connectivity	1	0	2	1	0	-	-	-	-	
		E. Bike Lanes	1	0	2	1	0	-	-	-	-	
		F. Bike Parking	1	0	2	1	0	-	-	-	-	
		G. Bike-Pedestrian Trails-Paths	1	0	2	1	0	-	1	-	0	
		H. Other Walkability	1	0	2	1	0	-	-	-	-	
		I. Mixed Use	1	0	2	1	0	2	1	-1	0	
		J. Active Recreation	1	0	2	1	0	-	1	-	0	
		K. Passive Recreation	1	0	2	1	0	-	1	-	0	

Y	N	5. Planned Unit Development (PUD) Category	Addressed?		Strength			Type of Use				Citation(s)
			Yes	No	Req	Enc	No	Mixed	Allow	Prohib	None	
		A. Sidewalks	1	0	2	1	0	-	-	-	-	
		B. Crosswalks	1	0	2	1	0	-	-	-	-	
		C. Bike-Pedestrian Connectivity	1	0	2	1	0	-	-	-	-	
		D. Street Connectivity	1	0	2	1	0	-	-	-	-	
		E. Bike Lanes	1	0	2	1	0	-	-	-	-	
		F. Bike Parking	1	0	2	1	0	-	-	-	-	
		G. Bike-Pedestrian Trails-Paths	1	0	2	1	0	-	1	-	0	
		H. Other Walkability	1	0	2	1	0	-	-	-	-	
		I. Mixed Use	1	0	2	1	0	2	1	-1	0	
		J. Active Recreation	1	0	2	1	0	-	1	-	0	
		K. Passive Recreation	1	0	2	1	0	-	1	-	0	

Y	N	6. Public, Civic, Gov't Districts Category	Addressed?		Strength			Type of Use				Citation(s)
			Yes	No	Req	Enc	No	Mixed	Allow	Prohib	None	
		A. Sidewalks	1	0	2	1	0	-	-	-	-	
		B. Crosswalks	1	0	2	1	0	-	-	-	-	
		C. Bike-Pedestrian Connectivity	1	0	2	1	0	-	-	-	-	
		D. Street Connectivity	1	0	2	1	0	-	-	-	-	
		E. Bike Lanes	1	0	2	1	0	-	-	-	-	
		F. Bike Parking	1	0	2	1	0	-	-	-	-	
		G. Bike-Pedestrian Trails-Paths	1	0	2	1	0	-	1	-	0	
		H. Other Walkability	1	0	2	1	0	-	-	-	-	
		I. Mixed Use	1	0	2	1	0	2	1	-1	0	
		J. Active Recreation	1	0	2	1	0	-	1	-	0	
		K. Passive Recreation	1	0	2	1	0	-	1	-	0	

Y	N	7. Residential Districts Category	Addressed?		Strength			Type of Use				Citation(s)
			Yes	No	Req	Enc	No	Mixed	Allow	Prohib	None	
		A. Sidewalks	1	0	2	1	0	-	-	-	-	
		B. Crosswalks	1	0	2	1	0	-	-	-	-	
		C. Bike-Pedestrian Connectivity	1	0	2	1	0	-	-	-	-	
		D. Street Connectivity	1	0	2	1	0	-	-	-	-	
		E. Bike Lanes	1	0	2	1	0	-	-	-	-	
		F. Bike Parking	1	0	2	1	0	-	-	-	-	
		G. Bike-Pedestrian Trails-Paths	1	0	2	1	0	-	1	-	0	
		H. Other Walkability	1	0	2	1	0	-	-	-	-	
		I. Mixed Use	1	0	2	1	0	2	1	-1	0	
		J. Active Recreation	1	0	2	1	0	-	1	-	0	
		K. Passive Recreation	1	0	2	1	0	-	1	-	0	

Copyright © 2013 The Board of Trustees of the University of Illinois

Y	N	8. General Zoning Provisions		Addressed?			Strength				Type of Use					Citation(s)
				Yes	No		Req	Enc	No		Mixed	Allow	Prohib	None		
		A. Sidewalks		1	0		2	1	0		-	-	-	-		
		B. Crosswalks		1	0		2	1	0		-	-	-	-		
		C. Bike-Pedestrian Connectivity		1	0		2	1	0		-	-	-	-		
		D. Street Connectivity		1	0		2	1	0		-	-	-	-		
		E. Bike Lanes		1	0		2	1	0		-	-	-	-		
		F. Bike Parking		1	0		2	1	0		-	-	-	-		
		G. Bike-Pedestrian Trails-Paths		1	0		2	1	0		-	1	-	0		
		H. Other Walkability		1	0		2	1	0		-	-	-	-		
		I. Mixed Use		1	0		2	1	0		2	1	-1	0		
		J. Active Recreation		1	0		2	1	0		-	1	-	0		
		K. Passive Recreation		1	0		2	1	0		-	1	-	0		

D. Complete Streets Policy

COMPLETE STREETS POLICY		Addressed?	
		Yes	No
Complete Streets Policy Addressed?		1	0

Complete Streets Policy Coded	
County	1
Place	2
Place and County	3

Strength				CS Policy Adoption Date		Citation(s)
Req	Enc	No				
2	1	0		__ __ / __ __ / __ __ __ __		

E. Record Notes

Notes for this record:

Form Status	
Incomplete	0
Unverified	1
Complete	2

Copyright © 2013 The Board of Trustees of the University of Illinois
